# Mucosal eosinophilic infiltration may be a characteristic of human intestinal spirochetosis

**DOI:** 10.1186/s12879-021-06418-8

**Published:** 2021-07-31

**Authors:** Sho Ogata, Ken Shimizu, Susumu Tominaga, Susumu Matsukuma

**Affiliations:** 1 Department of Pathology and Laboratory Medicine, National Defense Medical College, Tokorozawa, Saitama 359-8513 Japan; 2 Department of Diagnostic Pathology, JCHO Saitama Medical Center, Saitama, Saitama 330-0074 Japan; 3 Department of Laboratory Medicine, National Defense Medical College Hospital, Tokorozawa, Saitama 359-8513 Japan

**Keywords:** *Brachyspira*, Eosinophil, Human intestinal spirochetosis, Spirochete

## Abstract

**Background:**

Human intestinal spirochetosis (HIS) is an infectious disease of large intestines caused by *Brachyspira* species, and most HIS cases are asymptomatic or exhibit mild intestinal symptoms. The host reaction to HIS remains unclear, and we examined HIS-related mucosal inflammatory features histologically.

**Methods:**

From the archival HIS cases in a single medical center, 24 endoscopically taken specimens from 14 HIS cases (male:female = 10:4; 28–73 yrs) were selected as not containing polypoid or neoplastic lesions. Stromal neutrophils, eosinophils, and mast cells, and intraepithelial neutrophils and eosinophils, (sNeu, sEo, sMast, iNeu, and iEo, respectively) were counted, and the presence or absence of lymphoid follicles/aggregates (LFs) was also examined. Association of the above inflammation parameters and spirochetal infection parameters (such as degrees of characteristic fringe distribution, of spirochetal cryptal invasion, and of spirochetal intraepithelial invasion) were also analysed.

**Results:**

iNeu was observed in 29.2%, iEo in 58.3%, and LFs in 50.0% of the specimens. Maximal counts of sNeu, sEo, sMast, iNeu, and iEo averaged 8.4, 21.5, 6.0, 0.5 and 1.5, respectively. Strong correlation between the maximum counts of iNeu and iEo (*p* < 0.001, *r* = 0.81), and correlations between those of iEo and sNeu (*p* = 0.0012, *r* = 0.62) and between those of iEo and sEo (*p* = 0.026, *r* = 0.45) were observed. iNeu was influenced by fringe formation (*p* < 0.05) and spirochetal crypt involvement (*p* < 0.05).

**Conclusions:**

HIS was accompanied by inflammatory reactions, and among these, mucosal eosinophilic infiltration may be a central indicator and host reaction of HIS.

## Background

Human intestinal spirochetosis (HIS) is an infectious disease of the large intestine caused by *Brachyspira* species. Most HIS cases are asymptomatic or exhibit only mild intestinal symptoms (such as mild diarrhea or a slight increase in bowel habits) [1, 2], and thus HIS might be considered non-harmful [1, 3]. Therefore, most cases are found *incidentally* by histological examination of specimens taken endoscopically during (a) annual check-up in healthy persons, (b) further investigation after positive results for fecal occult blood, or (c) follow-up after polypectomy/surgery for tumorous lesions [2]. However, some HIS cases manifest a significant degree of mucosal inflammation [4, 5] or exacerbated inflammaton of accompanying ulcerative colitis [6], and thus the pathogenesis of HIS is considered uncertain. Histologically, mucosal inflammation in HIS has been reported to be mild or non-specific [1], but mucosal eosinophilia was indicated in a recent report [7]. In the present study, we examined both intraepithelial and stromal inflammation in HIS-involving large intestines using endoscopically taken specimens, and found evidence that mild but significant degrees of inflammation can exist in HIS.

## Methods

**Fig. 1:**
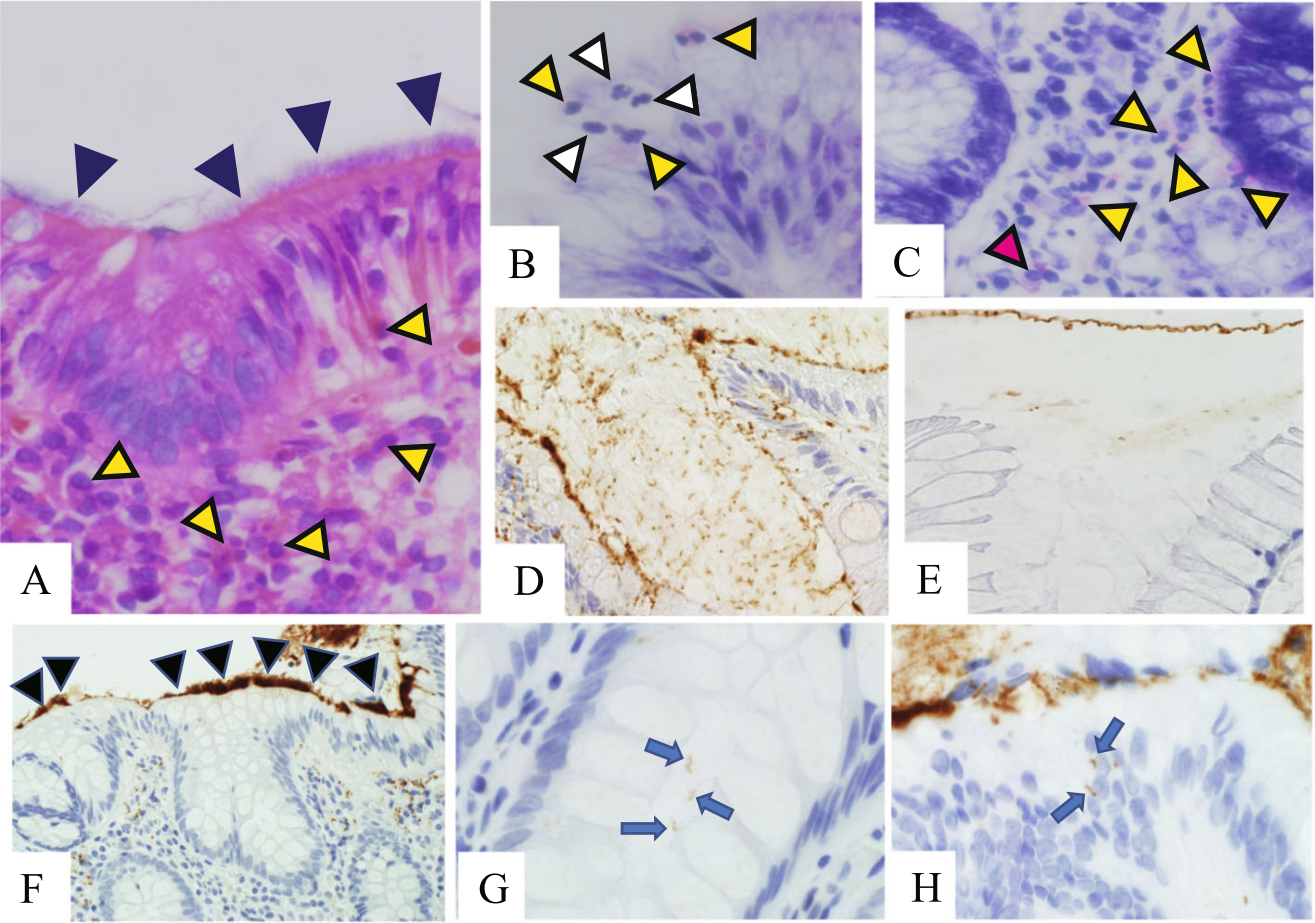
Histology of HIS specimens. **A-C**. Histology of hematoxylin & eosin (**A**) and Giemsa-stained (**B** & **C**) sections revealed neutrophils (white arrowheads), eosinophils (yellow arrowheads), and a metachromatic mast cell (red arrowhead in **C**). Histology also displayed hematoxylinophilic structures (i.e., fringes) covering the surface epithelium (black arrowheads in **A**). **D-H** Immunohistochemistry using anti-*Treponema pallidum* antibody showed many immunopositive spiral bacteria within the attached mucus and epithelial layer in HIS samples (**D**: positive control section). This was almost negative and no spiral bacteria were evident in non-HIS samples (**E**: negative control section). Immunohistochemistry revealed thick, band-like fringes on the surface epithelium (black arrowheads in **F**) and spiral organisms either within the crypt lumens (blue arrows in **G**) or within the surface epithelial layers (blue arrows in **H**). **A**: hematoxylin & eosin, × 400; **B** & **C**: Giemsa, × 400; **D-F**: diaminobenzidine, **D** × 200, **F** × 100, **E**, **G** & **H** × 400

We reviewed endoscopically taken specimens that had been histologically diagnosed with HIS. These materials were HIS cases that were archived at the Department of Diagnostic Pathology, JCHO Saitama Medical Center (Saitama, Japan), having been detected in three separete years (2001, 2006, and 2011). Clinical information and colonoscopic features were collected from the pathology request forms, although data concerning eosinophil numbers in the peripheral blood count were not collected in the patients providing specimens used in the present study. Using hematoxylin-eosin (HE)-stained sections, cases providing one or more specimens that histologically exhibited a distinct, hematoxylinophilic fringe-formation on the luminal surface of the colorectal surface epithelium (arrowheads in Fig. 1a) were considered to have HIS [2]. Among these, the sections reviewed here were limited to those displaying non-polypoid and non-neoplastic lesions (viz. those likely to be diagnosed as “inflamed mucosae”). All sections from these HE-indicated HIS cases were further examined using Giemsa-stain and immunohistochemistry (IHC). A total of 24 specimens from 14 HIS cases (male:female = 10:4; median age, 52 years; 28–73 years) was examined.

In the present study, we focused on inflammation parameters, namely: (1) stromal infiltrates of neutrophils, eosinophils, and mast cells (sNeu, sEo, and sMast, respectively), (2) intraepithelial infiltrates of neutrophils and eosinophils (iNeu and iEo, respectively), and (3) lymphoid follicles/aggregates (LFs). These cells were counted in each high power field (HPF; objective × 40 and field number 22 mm; viz. 0.55 mm in diameter), and the maximum values in each specimen were obtained. Neutrophils and eosinophils were counted using both HE and Giemsa-stained sections (Figs. 1A and B), while mast cells were counted using Giemsa-stained sections (Fig. 1C). sNeu, sEo, and sMast were counted in each type of cell in the lamina propriae, and iNeu and iEo in the surface epithelial layers. Findings concerning LFs were divided into two groups (presence or absence).

For IHC, we applied *Treponema pallidum* (*TP*) antibody to detect *Brachyspira* in the specimens of HIS cases, as reported previously [8, 9] (Figs. 1D and e). We performed the polymer-peroxidase method [Histofine® Simple Stain MAX PO 3 (MULTI); Nichirei Bioscience, Tokyo, Japan] on deparaffinized sections from HIS cases. After pretreatment with the heat-induced epitope retrieval technique, the polyclonal antibody for *TP* (Abcam, UK) was incubated for half an hour [9]. In the evaluation of IHC, we considered thick and feathery immunopositive bands on the surface epithelium to be fringes (Fig. 1F). Immunoreactive fringes and spiral organisms within the crypt lumens (Fig. 1G) and within the surface epithelial layers (Fig. 1H) were also investigated, as follows [9]: (a) the mucosal surface coverage with “fringes” [(−) absence of fringes, (+) < 50% of the entire surface mucosa covered by fringe, or (2+) > 50% covered by fringe], (b) spiral organisms within crypts [(−) absence of spiral organisms, (+) if a few crypts in the superficial half of the mucosa contained them, or (2+) if crypts in the deeper half of the mucosa or several crypts contained them], and (c) intraepithelial spiral organisms [(−) absence or (+) presence]. These spirochetal infection parameters were analysed for their correlations with the inflammation parameters mentioned above.

In the statistical analysis, for which JMP pro version 14.0.0 software (SAS Institute, Inc., Cary, NC) was employed, Pearson’s correlation coefficient test was performed to analyse correlations among inflammation parameters, while the Wilcoxon rank sum test was used to analyse correlations between the various inflammation parameters and spirochetal infection parameters or colonoscopic features, and two tailed Fisher’s exact probability test for between the presense or absence of LFs and colonoscopic features, with *p* < 0.05 being considered significant. This study was approved by the local ethics committee of the JCHO Saitama Medical Center [Approval number: No. 15–11 (July 9, 2015)].

## Results

### Patient profiles and colonoscopic features

Of the 14 examined HIS cases, five exhibited gastrointestinal symptoms such as diarrhea, soft stools, or frequent bowel habits, and one of these was a single case of suspected irritable bowel syndrome. The other nine cases were asymptomatic; among these, four cases had undergone colonoscopy in their annual check-up, one for cancer surveillance for an elevation in serum carcinoembryonic antigen value, and four for polypectomy or follow-up after colectomy. Colonoscopic findings for each site from which the 24 specimens were taken were 17 reddish/erosive, 1 edematous, and 6 normal mucosae.

### Inflammation parameters

**Fig. 2:**
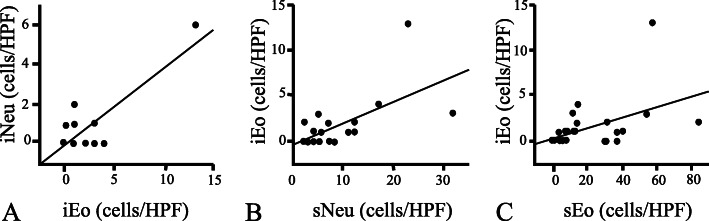
Correlation between eosinophil counts and neutrophil counts. Intraepithelial eosinophil count (iEo) was strongly correlated to intraepithelial neutrophil counts (iNeu) (*p* < 0.001, *r* = 0.81; **A**). iEo was also correlated both to stromal neutrophil count (sNeu) (*p* = 0.0012, *r* = 0.62; **B**) and stromal eosinophil count (sEo) (*p* = 0.026, *r* = 0.45; **C**). HPF: high power field

In the lamina propriae, the maximum neutrophil, eosinophil, and mast cell counts/HPF averaged 8.4 (2–32), 21.5 (0–84), and 6.0 (1–18), respectively. Within the surface epithelial layer, iNeu was observed in 7 specimens (29.2%) and iEo in 14 specimens (58.3%), with the maximum cell counts/HPF averaging 0.5 (0–6) and 1.5 (0–13), respectively. Statistics revealed a strong correlation between the maximum cell counts/HPF of iNeu and iEo (*p* < 0.001, *r* = 0.81; Fig. 2A), and correlations between those of iEo and sNeu (*p* = 0.0012, *r* = 0.62; Fig. 2B) and between those of iEo and sEo (*p* = 0.026, *r* = 0.45; Fig. 2C). LFs were observed in 12 specimens (50.0%). However, there were no significant associations between the values obtained for sNeu, sEo, sMast, iNeu, iEo, and presence/absence of LFs.

Analysis of associations among the above inflammatory parameters and the corresponding colonoscopic features was also performed. Averaged, maximum cell counts/HPF in specimens taken from endoscopically reddish/erosive mucosae did not differ from those in edematous/normal mucosae, as follows: in reddish/erosive mucosae vs. in edematous/normal mucosae: 0.71 (0–2) vs. 0.1 (0–1) for iNeu (*p* = 0.09), 1.8 (0–13) vs. 0.9 (0–2) for iEo (*p* = 0.92), 9.7 (2–32) vs. 5.3 (2–7) for sNeu (*p* = 0.22), 18.0 (2–57) vs. 30.0 (0–84) for sEo (*p* = 0. 46), and 4.6 (1–14) vs. 9.4 (2–18) for sMast (*p* = 0.11). LFs were observed in 7 (of 17) specimens (41.1%) from the reddish/erosive mucosae and in 5 of 7 (71.4%) in those from the edematous/normal mucosae, and there was no difference between the presence or absence of LFs and colonoscopic features (*p* = 0.64).

### Relationship between inflammation parameters and spirochetal infection parameters

Concerning spirochetal infection parameters, the percentage of specimens placed in the various classes were as follows: (−) 8 specimens (33%), (+) 4 (17%), (2+) 12 (50%) for fringe distribution; (−) 17 (71%), (+) 4 (17%), (2+) 3 (12%) for spirochetal crypt involvement; and (−) 7 (29%) and (+) 17 (71%) for intraepithelial spirochetal involvement.

**Fig. 3:**
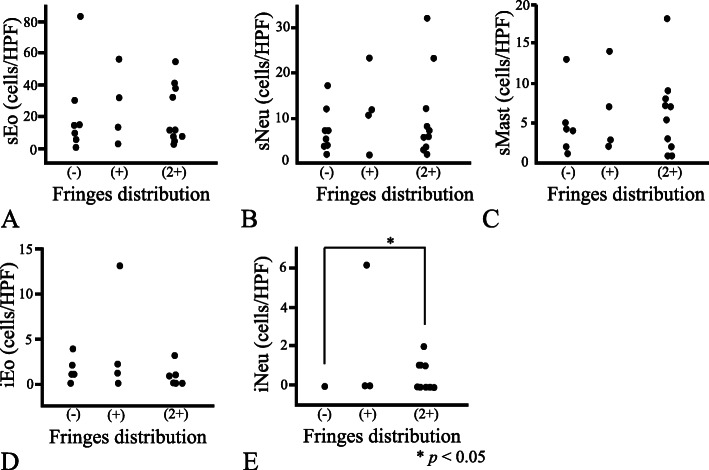
Relationship between degree of fringe distribution and the various inflammation parameters. Specimens were classified into three groups, as follows: (−) absence of fringes, (+) < 50% of the entire surface mucosa covered by fringe, and (2+) > 50% covered by fringe. The degree of fringe distribution in the specimens did not affect the values obtained for stromal eosinophil, neutrophil, or mast cell count (sEo, sNeu, and sMast, respectively) (**A-C**). The same spirochetal invasion parameter did not influence the value obtained for intraepithelial eosinophil count (iEo) (**D**), but presence of fringe did increase the intraepithelial neutrophil count (iNeu) [**E**; fringe (−) vs. fringe (2+), *p* = 0.024]. HPF: high power field

**Fig. 4:**
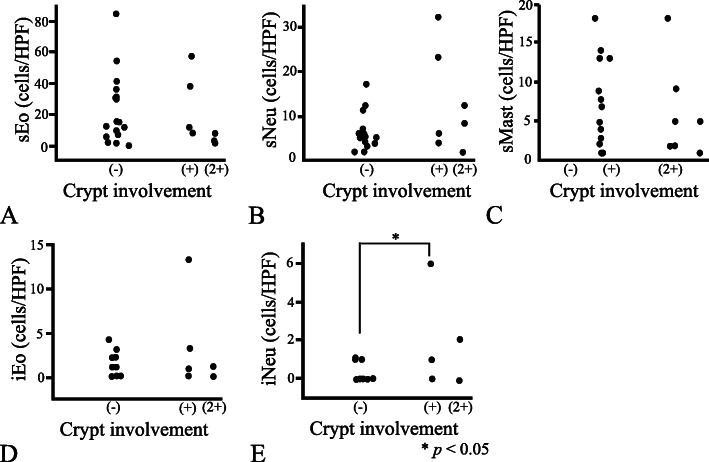
Relationship between degree of spirochetal crypt involvement and the inflammation parameters. Specimens were classified into three groups, as follows: (−) absence of spiral organisms, (+) if a few crypts in the superficial half of the mucosa contained them, (2+) if crypts in the deeper half of the mucosa or several crypts contained them. The degree of spirochetal involvement within crypt lumens in the samples did not affect the values obtained for stromal eosinophil, neutrophil, or mast cell count (sEo, sNeu, and sMast, respectively) (**A-C**). The same spirochetal invasion parameter did not influence the intraepithelial eosinophil count (iEo) (**D**), but spirochetal presence within crypt lumens did increase the intraepithelial neutrophil count (iNeu) [**E**; crypt (−) vs. crypt (+), *p* = 0.02]. HPF: high power field

**Fig. 5:**
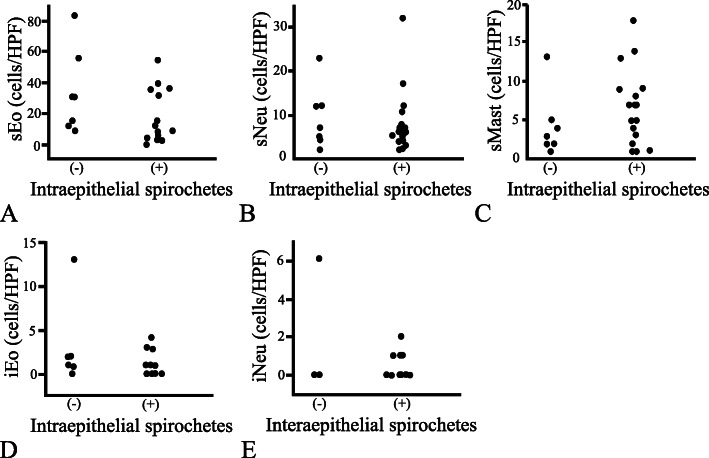
Relationship between presence/absence of intraepithelial spirochetal invasion and the inflammation parameters. Specimens were classified into two groups, as follows: (−) absence or (+) presence of spirochetes in the surface epithelial layer. The presence or absence of intraepithelial spirochetes in the samples did not affect the values obtained for stromal eosinophil, neutrophil, or mast cell count (sEo, sNeu, and sMast, respectively) (**A-C**), nor the intraepithelial eosinophil or neutrophil count (iEo and iNeu, respectively) (**D** & **E**). HPF: high power field

Regarding the inflammation parameters, the numbers obtained for stromal inflammatory infiltrates (sNeu, sEo, and sMast) did not differ with any of the spirochetal infection parameters (viz. the degree of fringe distribution, spirochetal crypt involvement, or intraepithelial spirochetal invasion) (Figs. 3, 4 and 5). Likewise, iEo did not differ with any of these spirochetal parameters. Although iNeu did not differ with the degree of intraepithelial spirochetal invasion, it was influenced both by fringe formation (*p* < 0.05) and by spirochetal crypt involvement (*p* < 0.05) (Figs. 3E & 4E). Whether LFs were present or absent was not significantly associated with any of the spirochetal infection parameters (Figures not shown).

## Discussion

The present study of HIS revealed: 1) a relatively high sEo count, 2) the presence of iNeu and iEo, and 3) the presence of LFs. In part, the above findings paralleled the results of a Swedish report in which biopsy samples from HIS cases revealed the presence of both mucosal eosinophilia and lymphoid follicles [7]. In that Swedish study, the authors showed mild mucosal eosinophilia (mean 30 cells/mm^2^) in 17 HIS cases and also suggested an association of HIS with irritable bowel syndrome [7]. However, HIS may be present in specimens containing polypoid or neoplastic lesions, such as conventional adenomas or hyperplastic polyps [5], and the 17 HIS cases in the Swedish study included 3 cases having tubular adenomas, 3 hyperplastic polyps, and 2 diverticular disease. These accompanying lesions may be considered to modify the cell types and numbers present. Thus, in the present study, we excluded specimens containing polypoid or neoplastic lesions. Nevertheless, even using these selected specimens, we could confirm the results of the Swedish study [7].

First, the average sEo value in the present specimens was slightly over 20 cells/HPF, which possibly meets the histologic criteria for eosinophilic gastroenteritis (EGE) [10], although the eosinophil number in *normal* colonic mucosae remains in discussion [10]. EGE is characterized by an accumulation of eosinophils in the digestive tract, and sometimes requires histologic confirmation. The pathogenesis of EGE is thought to be related to a hypersensitivity reaction, and by radiology and endoscopy, mucosal edema may be seen in EGE [11]. Interestingly, marked mucosal edema has been reported in HIS, too [12]. Concerning EGE criteria, accompanying infectious disease should be excluded from its diagnosis [11]. However, from the results of the present study, some HIS cases may possibly be diagnosed *incorrectly* to be EGE because a histologic sign of HIS (viz. the fringe formation on the mucosal surface) may be too subtle or focal for it to be easily recognized, even by experienced pathologists. Moreover, the diagnostic fringes are not always found everywhere in the large intestine of HIS cases [13] and we noted that stromal or intraepithelial eosinophil infiltration (sEo or iEo, respectively) was not influenced by the degree of fringe distribution. In the present study, intraepithelial eosinophil counts were related not only to their stromal counts, but also to both the intraepithelial and stromal *neutrophil* counts. Thus, stromal and intraepithelial eosinophilic infiltration might be a central indicator of HIS and a host reaction to HIS.

HIS might be underestimated, indeed considered a harmless condition that is not worthy of being called a “disease”, since spirochetal residence may be part of the normal flora in human large intestines [1, 3]. However, neutrophilic infiltration, especially in the surface epithelial layer, generally indicates active mucosal inflammation, and its presence in HIS indicates that HIS exhibits histologic signs of “infectious disease”. In particular, iEo was observed in more than half the present specimens. In the present study, we found that neutrophilic and eosinophilic infiltration into the surface epithelial layer (viz. iNeu and iEo, respectively) were strongly correlated, while our data suggesting that intraepithelial neutrophil infiltration, at least, might be a reaction to spirochetal attachments on the surface epithelium and/or spirochetal presence in crypt lumens. In other words, these spirochetal burdens might be thought sufficient stimuli to induce intraepithelial neutrophilic or eosinophilic infiltration. However, these active inflammation parameters were not influenced by whether intraepithelial spirochetal invasion was or was not present. One possibility to consider is that spirochetal invasion into the epithelial layer might not be destructive or cytotoxic to the surface epithelium, but simply permeative (mainly between the cell borders of intact or apoptotic surface epithelium). If that is correct, spirochetal invasion might not add further stimuli to those due to the spirochetal burden on the epithelial surface and/or crypt lumens. Similarly, the degree of eosinophilic activation and tissue damage were presumably not severe enough to increase mast cells since the mast cell count in the present study was not related to sEo values, and moreover remained lower or similar to the basal level of mast cells shown in previous reports [14, 15].

In the present study, LFs were observed in half of all specimens from HIS cases. These findings suggest the presence of HIS-related chronic mucosal inflammation, although the presence of LFs did not influence eosinophils, neutrophils, or mast cells counts or the spirochetal infection parameters that we examined. In our previous immunohistochemical investigation using surgically removed specimens with HIS, possible spirochetal fragments were detected within LFs [13]. On that basis, LFs might be another immunologic host reaction to a spirochetal burden.

The present study has some limitations regarding its materials: (1) we analyzed a small number of HIS cases that were archived and that were detected at only a single hospital (although it is a medical center in a location neighboring Tokyo, the capital of Japan, and specimens were also collected from other hospitals in the same prefecture) in only three calendar years, (2) potential inaccuracy may have resulted from IHC cross-reaction with the antibody used in the present study (antibodies specific for *Brachyspira* species not being commercially available) and we did not perform further confirmation, such as species genotyping or *in situ* hybridization (cases were limited to those exhibiting *distinctive* fringes), and (3) the inflammation parameters were limited to a few cell types, and excluded lymphocytes, plasma cells, and macrophages (these cells, especially macrophages, might contribute to the mucosal immunity to tissue-invading spirochetes [13], although examining them was beyond the scope of present study).

## Conclusions

Histologically, HIS was accompanied by inflammatory reactions, including eosinophilia, lymphoid follicles/aggregates, and intraepithelial neutrophils and eosinophils (to varying degrees). Stromal and intraepithelial eosinophilic infiltration might be a central indicator of HIS and a host reaction to HIS.

## Data Availability

The datasets generated and/or analysed during the current study are not publicly available due to protection of potentially contained patient health information in a single hospital.

## Abbreviations

HIS: Human intestinal spirochetosis
sNeu: stromal neutrophil
sEo: stromal eosinophil
sMast: stromal mast cell
iNeu: intraepithelial neutrophil
iEo: intraepithelial eosinophil
LF: Lymphoid follicles/aggregates
HE: Hematoxylin-eosin
TP: *Treponema pallidum*
IHC: Immunohistochemistry
HPF: High power field
EGE: Eosinophilic gastroenteritis
